# Clinical usefulness of objective tongue image devices monitoring in temporal tongue color: a demonstration of successively treated cases with traditional medicines

**DOI:** 10.3389/fmed.2026.1923285

**Published:** 2026-09-21

**Authors:** Aya Murakami, Takao Namiki, Atsushi Ishige, Sadayuki Ochi, Monika Suzuki, Akira Morita, Tatsuro Katsuno, Akihide Sumino, Kohsuke Hayamizu, Toshiya Nakaguchi

**Affiliations:** 1 Laboratory of Food Chemistry, Yokohama University of Pharmacy, Yokohama, Japan; 2 Sumida Kampo Clinic, Chiba University Hospital, Tokyo, Japan; 3 Center for Preventive Medicine, International University of Health and Welfare, Narita Hospital, Chiba, Japan; 4 Yokohama University of Pharmacy, Yokohama, Japan; 5 Department of Health Pharmacy, Yokohama University of Pharmacy, Yokohama, Japan; 6 Department of Medical Engineering, Graduate School of Science and Engineering, Chiba University, Chiba, Japan; 7 Center for Frontier Medical Engineering, Chiba University, Chiba, Japan

**Keywords:** Kampo medicine, tongue color, tongue diagnosis, tongue image analyzing system, traditional Japanese medicine

## Abstract

**Background:**

In Kampo medicine (traditional Japanese medicine), the diagnostic method of observing a patient's tongue is called the ‘tongue diagnosis’. Information provided by observation of a patient's tongue is used to help select prescriptions at the initial consultation, monitor treatment effectiveness after therapy begins, and determine whether prescriptions should be changed. Kampo practitioners must therefore accurately compare the patient's past tongue condition with the current condition. However, it can be difficult to remember the precise details regarding a patient's previous tongue's appearance and to consistently make accurate comparisons. Given the need for objective and reproducible method of documenting tongue findings, we investigated the usefulness of our developed Tongue Image Analyzing System (TIAS). The application of the TIAS for two representative patients is described herein.

**Methods:**

We retrospectively reviewed patients who underwent tongue photography after providing informed consent and selected two cases with contrasting degrees of longitudinal changes in tongue findings. One patient demonstrated subtle changes in tongue color that were difficult to detect with the naked eye, and the other patient showed marked tongue color changes accompanied by changes in the heterogeneity of the tongue coating. We obtained the tongue color values in each case from tongue images photographed by TIAS as RGB data and converted into the CIE L*a*b* color space.

**Results:**

The tongue color of case1 was analyzed at three time points. The color difference from the first consultation (calculated as *ΔE*_00_ using L*a*b* values) increased by *ΔE*_00_ 1–2 after 42 days. This change was too subtle to be perceived by the naked eye. The tongue color of case 2, tongue color was analyzed at five time points. The color difference between the first consultation and 105 days later was *ΔE*_00_ 10.08, indicating a substantial change in tongue color.

**Conclusion:**

TIAS enables standardized longitudinal tongue imaging and facilitates the objective and quantitative comparison of subtle changes in tongue color that may be difficult to discern visually. These findings suggest that TIAS may be useful for objectively documenting and comparing longitudinal changes in tongue findings in clinical practice.

## 1 Introduction

The tongue is an organ covered by mucosal tissue and contains many blood vessels as well as minor salivary glands, including the glands of von Ebner. Visual observation of the tongue has thus been considered useful for assessing patients’ microcirculation and gastrointestinal function (1, 2). In the nineteenth century, Western physicians recognized the clinical value of a tongue examination and incorporated it into routine medical practice (3). More recent and accumulating evidence has demonstrated associations between tongue findings and various diseases. In addition, monitoring of temporal tongue color has been reported to be useful for risk assessment and prognosis prediction in a wide range of conditions, including cancer, upper gastrointestinal disorders, diabetes mellitus, and cardiovascular diseases (4–8).

In Kampo medicine (traditional Japanese medicine), the diagnostic method of observing a patient's tongue is called the ‘tongue diagnosis’. Tongue diagnosis has a long history in traditional East Asian medicine, including Kampo medicine and traditional Chinese medicine. A tongue diagnosis enables a noninvasive assessment of physiological and pathological conditions, including the patient's constitution, blood circulation, and water metabolism (9, 10). Information obtained via a tongue diagnosis is used to support the selection of prescription medicines at the initial consultation, monitor treatment efficacy, and adjust the prescribed formula as needed (11, 12). The effective utilization of tongue diagnosis requires the accurate evaluation of changes in the tongue body's color and the tongue coating's color, and consequently, physicians must be able to compare a patient's current tongue findings with previous observations. However, human memory is inherently limited, which often makes it difficult for physicians to consistently recall and accurately compare the historical tongue findings of their patients across the passage of time.

To address this limitation, various devices have been developed worldwide to capture and record images of patients’ tongues for diagnostic support (9, 13–15). However, tongue images obtained using these devices often suffer from reproducibility issues. This is because tongue colors are highly susceptible to environmental factors during observation (e.g., lighting conditions and ambient temperature), making it necessary to capture images under identical conditions each time (16). Figure 1 provides photographs of the same tongue taken simultaneously under different imaging conditions or using different digital photograph devices (smartphones). Not only does the perceived tongue color vary among the images; the skin color also appears different. These findings illustrate the difficulty of accurately documenting longitudinal changes in tongue color. Despite the importance of eliminating factors that may mislead clinical interpretation, to the best of our knowledge, only a very limited number of such devices have been reported.

**Figure 1:**
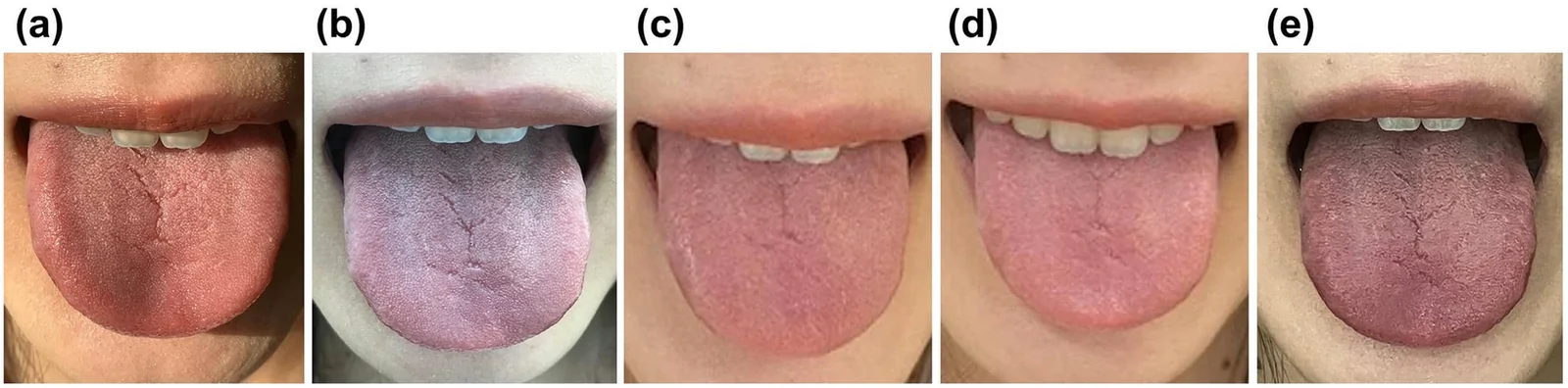
Different locations and methods of photographing results in different colors and skin tones. **(a)** Beside the window. **(b)** Window with the blinds closed under fluorescent lights. **(c–e)** Three different types of smartphones. Five tongues were photographed at same time, and these were same individual.

Tongue Image Analyzing System (TIAS) enables photography of the tongue under consistent conditions (Figure 2A). By maintaining a constant distance between the patient's tongue and the camera and employing an integrating sphere with diffuse illumination, TIAS provides a uniform light intensity and allows tongue images to be acquired under stable conditions that are unaffected by environmental factors such as light source characteristics, dryness, and ambient temperature (17–19). TIAS enables the acquisition of tongue images in which the gloss component of the tongue surface –– a factor that affects color reproducibility — has been eliminated. In addition, by using the tongue images captured by TIAS, it is possible to quantify the diameter of the sublingual vein, tongue surface characteristics, and the tongue color values using the L*a*b* color system defined by the Commission internationale de l'éclairage (CIE).

**Figure 2:**
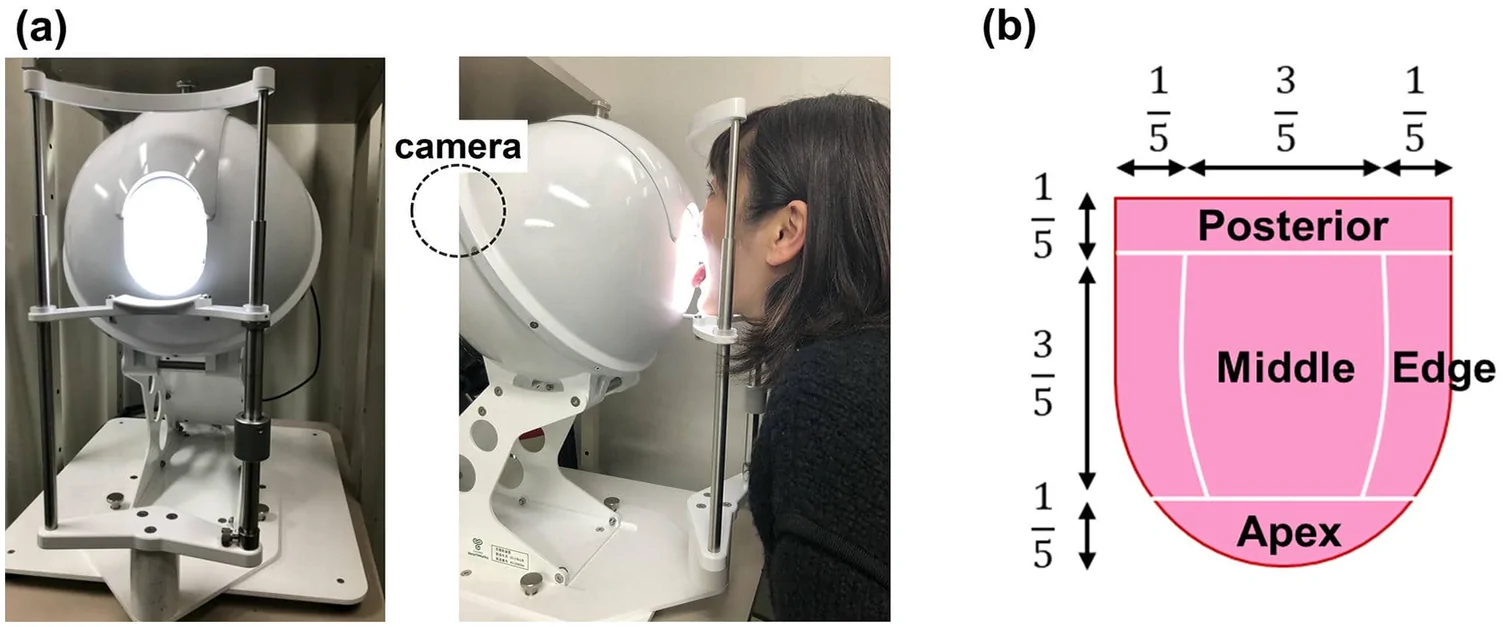
Tongue image analyzing system (TIAS). **(a)** Tongue image acquisition using TIAS. The patient's tongue was positioned using a chin rest, and tongue images were acquired under standardized imaging conditions. **(b)** TIAS quantifies tongue color values in four predefined regions based on the organ system patterns used in Kampo medicine: the tongue edge, tongue posterior, tongue middle, and tongue apex.

To eliminate bias and ensure objectivity, clinical examination methods are generally standardized and examination results are expressed as quantitative values. In addition to enabling tongue imaging and recording under consistent conditions, TIAS provides objective documentation by quantifying the tongue's information such as color and characteristics (17–21). Furthermore, TIAS was developed to meet International Organization for Standardization (ISO) 20498-1－CTIS－Part 1; General requirement and ISO 20498-2－CTIS－part 2; Lighting environment. Our research of the relevant literature indicated that few studies have objectively and quantitatively evaluated treatment-associated changes in tongue color using ISO-compliant tongue imaging systems.

Given the need for a novel method of recording tongue findings, we present two cases in which tongue images were captured longitudinally using TIAS under standardized imaging conditions that minimize variations in illumination and other factors affecting tongue color measurements. The ability to record the tongue in an objective and reproducible manner may allow physicians to compare the tongue's appearance between the initial and follow-up visits without relying on memory or subjective impressions. With these two cases, we sought to illustrate the potential of TIAS to provide objective information that may assist physicians in clinical assessments and to quantify and compare subtle longitudinal changes in tongue color that may be difficult to appreciate by visual observation alone.

## 2 Methods

### 2.1 Quantitative evaluation of tongue colors using TIAS

TIAS was used for tongue imaging in both present cases (Figure 2A) (17–21). TIAS was developed to meet the requirements of ISO 20498-1 (CTIS—Part 1: General Requirements) and ISO 20498-2 (CTIS—Part 2: Lighting Environment), which address the standardization of the tongue imaging environment. Before the image acquisition in the present study, the device was calibrated using a color chart. A chin rest was used to standardize the positional relationship between the patient's tongue and the device. In addition, to standardize the imaging timing, each patient's first image (acquired approx. 1 s after tongue protrusion) was used for analysis at each imaging session.

Tongue color values were converted from RGB to L*a*b* color space data defined by the CIE based on tongue photographs taken with TIAS and used for analysis (8). The L*a*b* color space is a device-independent color space and a color system consisting of L* for lightness (brightness to darkness), a* for chromaticity (redness [ + a] to greenness [–a]), and b* for chromaticity (yellowness [ + b] to blueness [–b]). Furthermore, TIAS quantifies tongue color values in four regions: the tongue edge, tongue posterior, tongue middle, and tongue apex (17). Each region was defined by a ratio based on the tongue shape, as shown in Figure 2B.

In the present two cases, the CIEDE2000 color-difference formula (*ΔE*_00_) was calculated to quantify the changes in tongue color values (color differences) between the initial consultation and post-treatment time points (22). The *ΔE*_00_ is a metric that quantitatively expresses the color difference between two points in the CIEL*a*b* color space and has been widely applied in dental studies of teeth (23–25), gingiva (26–28), and the tongue (29, 30). In addition, in Case 2, we calculated the contrast in tongue surface characteristics in order to quantify the heterogeneity of the tongue coating.

### 2.2 Subjects

A retrospective analysis of the cases of 485 patients who visited the Department of Kampo Medicine at Chiba University Hospital between 2017 and 2023 was conducted. In our department, patients are fully informed at their initial visit about tongue imaging using TIAS and the use of tongue images for research purposes, and the patients’ consent for imaging is obtained at each subsequent visit. Tongue images were thus not necessarily obtained at every visit for all patients.

We included the case of patients for whom longitudinal tongue images, including an image obtained at the initial visit, were available and who underwent tongue imaging on at least three occasions. Among the patients meeting these selection criteria, we selected the case of two patients because they exhibited characteristic tongue colors and tongue findings and differed in the degree of longitudinal change in tongue color, thereby allowing a comparison of longitudinal changes in tongue findings to be illustrated (Figure 3).

**Figure 3:**
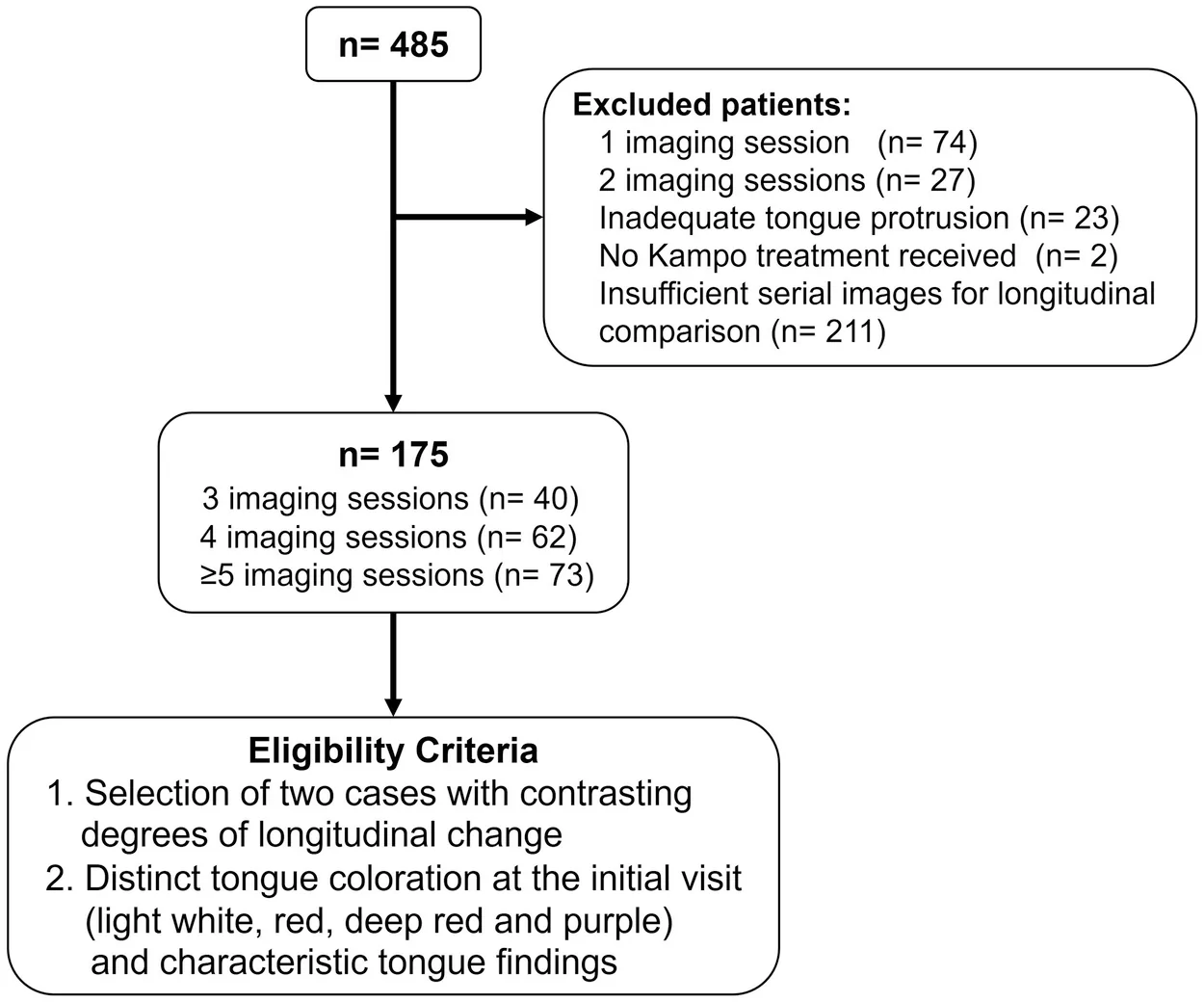
Patient selection process.

The first patient's case was chosen because it documented subtle changes in tongue color that were difficult to detect with the naked eye. The second patient's case was selected because it involved a conspicuous tongue color change and changes in tongue coating heterogeneity. Clinical information including each patient's physical findings, chief complaints, and Kampo prescriptions were extracted from the medical records.

In our department, patients are instructed on how to stick out their tongues before imaging and are given an opportunity to practice the procedure. During image acquisition, a chin rest is used to standardize the position of the patient's tongue relative to the TIAS.

The Department provided a thorough explanation of tongue photographing and patient consent was obtained at the time of the initial visit. This study was approved by the Ethics Committee of Chiba University (approval No. 812) and the patients’ anonymity was assured.

## 3 Results

### 3.1 Application case 1

#### 3.1.1 Clinical history

The case of patient 1 demonstrates subtle tongue color changes captured by TIAS that were difficult to detect with the naked eye.

A 28-year-old man presented with general fatigue, irritability, and stiff neck and shoulders. He had a history of childhood developmental disorders and was taking anxiolytic medication. At 19 years old, on top of the stress load from work, he began to show irritability and fatigue. In addition, he developed headaches compiled with a sensation of heaviness in the head when the weather was bad, such as typhoons. He suddenly became irritable even when there was nothing wrong, and he experienced a loss of motivation. At the initial consultation, he complained of thirst, preferred cold water, and frequently awoke at night because of thirst. He also experienced sudden sweating and was sensitive to heat. He had no history of smoking and was taking medication for hypertension and dyslipidemia.

At the initial consultation, the physician assessed the patient's tongue as a red tongue with tooth marks and slight swelling (Figure 4A). The physician prescribed Kampo Yokukansan [抑肝散] extract granules (29), which are used to regulate qi, and Kampo Goreisan [五苓散] extract granules (30), which are used to improve fluid disturbance. The patient's symptoms improved with these treatments, and the changes in his tongue color also improved (Figures 4B,C).

**Figure 4:**
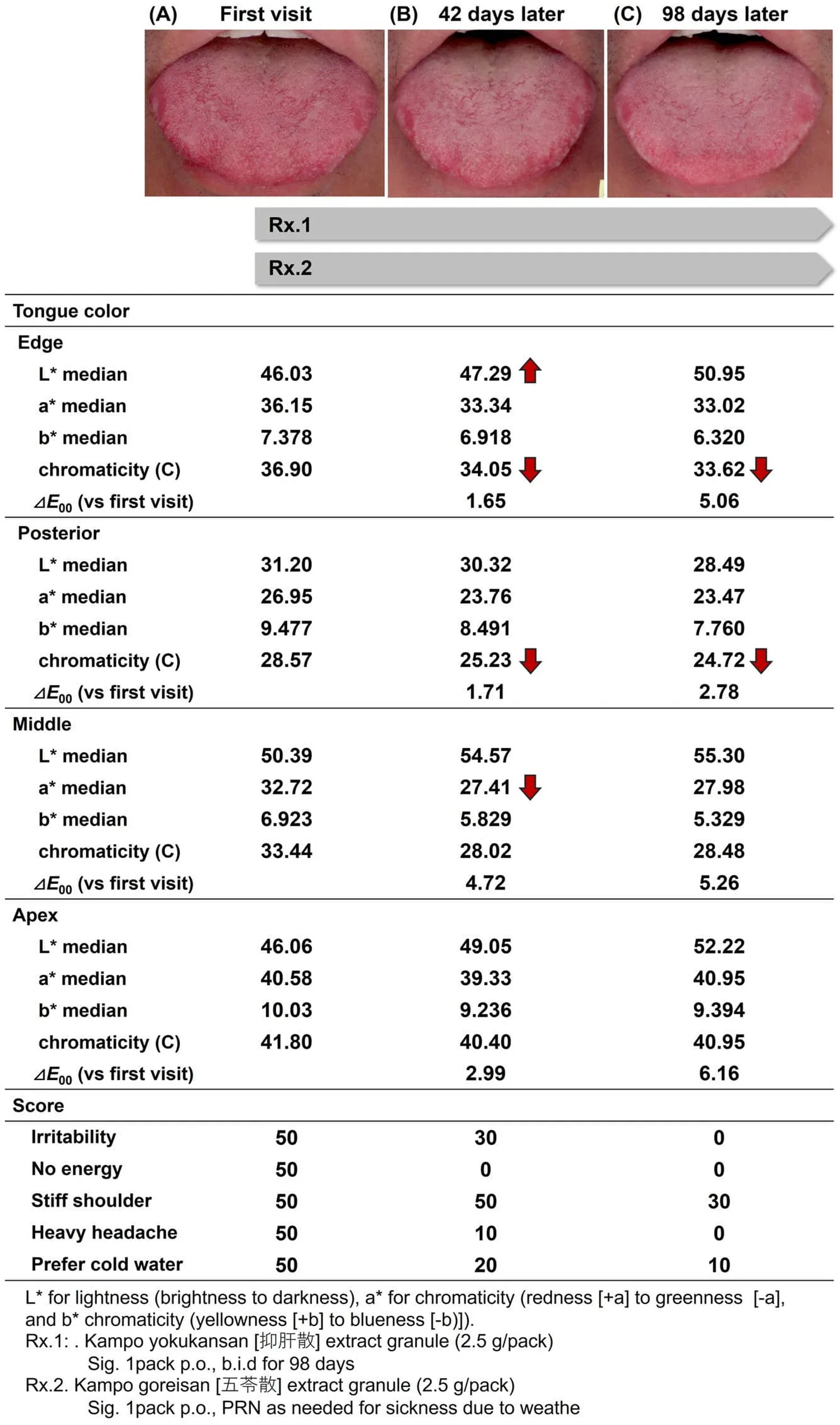
Application case 1, A 28-year-old man. (**A**) First visit, (**B**) 42 days, and (**C**) 98 days later starting Kampo treatment.

#### 3.1.2 Objective monitoring of the patient's tongue color changes

Figure 4 depicts the tongue images obtained at three time points for this patient at the initial consultation (Figure 4A), and after 42 days (Figure 4B), and after 98 days of the above-described Kampo treatment (Figure 4C), with the corresponding tongue color values in the figure's lower panel.

Relative to the initial consultation (Figure 4A), a reduction approximately 1－5 points in a* (chromaticity, redness) was observed in all of the tongue regions at 42 days treatment (Figure 4C), with the largest reduction of a* at the tongue middle area; from 32.7 to 27.41 points. The a* change between 42 days (Figure 4B) and 98 days (Figure 4C) was more subtle, as the a* values changed by approximately 0.3－1.6 points at all four regions, i.e., the tongue edge, tongue posterior, tongue middle, and tongue apex.

In the L*a*b* color space, chroma C (which represents color vividness) is calculated from the a* and b* values. Higher chroma values indicate greater color vividness. At 98 days of the Kampo treatment (Figure 4C), the chroma had decreased in all four tongue regions compared to the values recorded at the patient's initial consultation (Figure 4A), indicating a reduction in the overall vividness of the tongue color; at the tongue edge, the C value was 36.90 at the initial consultation, 34.05 at 42 days later, and 33.62 at 98 days later. The L* values (lightness) also increased in all four tongue regions.

To quantify changes in tongue color relative to the initial consultation, *ΔE*_00_ values were calculated. It has been reported that color differences with a *ΔE*_00_ value <1.5 are generally imperceptible to the human eye, and that such color differences become perceptible at *ΔE*_00_ > 3 and are readily noticeable at *ΔE*_00_ > 6 (31). From the present patient's initial consultation to 42 days later, differences in color were observed at the tongue edge (*ΔE*_00_ 1.65) and the tongue posterior (*ΔE*_00_ 1.75) revealing subtle tongue color changes that were difficult to perceive visually. Nevertheless, the chroma value decreased (tongue edge: C = 36.90 at the initial consultation vs. 34.05 at 42 days later) and lightness increased (tongue edge: L* = 46.03 at initial consultation vs. 47.29 at 42 days later), suggesting an improvement from a red tongue toward a light red tongue, which is considered the normal tongue color. The *ΔE*_00_ value at the patient's tongue apex was 2.99, representing a change that remained difficult to recognize visually; however, a similar decrease in chroma and an increase in lightness were observed at this region. In contrast, the comparison of the tongue images obtained at the initial consultation (Figure 4A) and 98 days later (Figure 4C) revealed *ΔE*_00_ values at approximately 5－6 across the four regions. The associated decrease in chroma and increase in lightness were readily apparent in the visual inspection of the tongue images.

### 3.2 Application case 2

#### 3.2.1 Clinical history

In this patient's case 2, marked changes in tongue color and tongue coating were objectively demonstrated by quantitative measurements. A 41-year-old woman presented with a burning sensation and pain in the oral cavity and tongue, as well as headaches. Four months prior to presentation, she had developed a burning sensation and pain affecting the oral cavity and tongue. She reported easy fatiguability and an 8 kg wight loss. In addition, she described experiencing severe headaches before menstruation that required analgesic medication. She complained of thirst and excessive sweating. Although she was prone to flushing and heat intolerance, she also experienced cold sensitivity, with cold hands and feet. She urinated more >10 times/ day, had normal bowel movements, and reported a poor appetite. She had no history of smoking and took acetaminophen once a week.

At the initial consultation, the physician assessed her tongue as a red tongue, geographic tongue, with slight swelling. The presence of geographic tongue was considered indicative of qi deficiency (energy deficiency) and disorder of the spleen, which in Kampo medicine is regarded as the organ system responsible for digestive function (9, 32). Accordingly, Kampo Keishikashakuyakuto [桂枝加芍薬湯] extract granules (33) were prescribed. In addition, the red tongue was interpreted as reflecting glossitis and an inflammatory condition; therefore, Kampo Orengedokuto [黄連解毒湯] extract granules (34), which are used to reduce inflammation, were prescribed. Following the initiation of treatment, the patient's tongue pain and appetite gradually improved.

#### 3.2.2 Objective monitoring of the patient's tongue color changes

Figure 5 presents the patient's tongue images acquired at the initial consultation (Figure 5A), and four time points during the treatment course, i.e., 14 (Figure 5B), 42 (Figure 5C), 70 (Figure 5D) and 105 days later (Figure 5E) after the initiation of Kampo treatment. The corresponding tongue color values and contrast measurements are shown in the lower panel of Figure 5. In this patient's case, the tongue posterior contrast was used as an index of tongue coating heterogeneity. Higher contrast values indicate greater surface heterogeneity. The contrast was highest at the patient's initial consultation (74.07), decreased 14 days later (57.45), and then increased slightly thereafter (67.64, 69.35, and 64.00, at 42, 70, and 105, of treatment respectively). These changes corresponded well with the patient's tongue images, in which geographic tongue was most evident at the initial consultation (Figure 5A), improved at Day 14 (Figure 5B), and reappeared mildly from Day 42 onward (Figures 5C,E).

**Figure 5:**
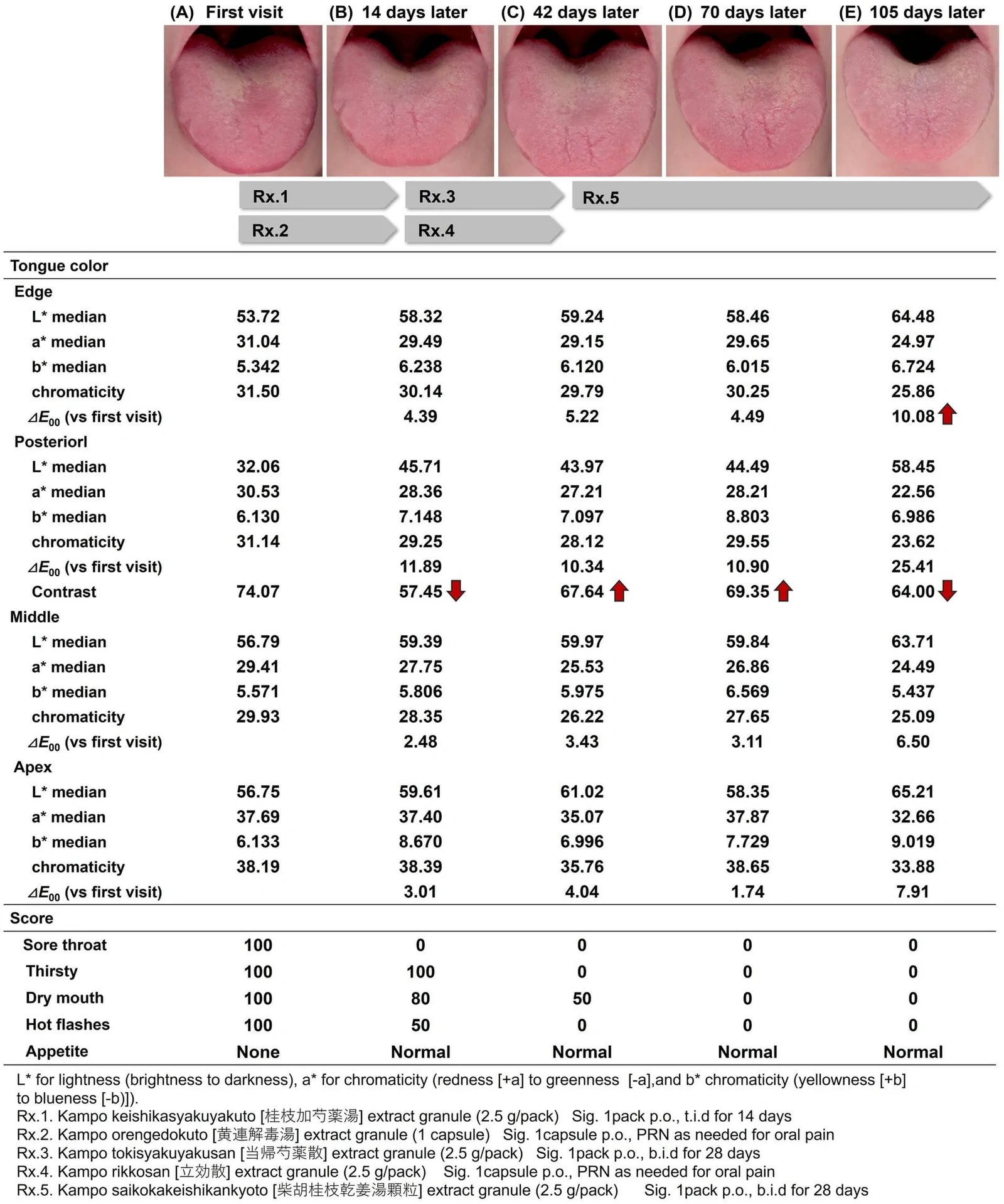
Application case 2, A 41-year-old woman. (**A**) First visit, (**B**) 14 days, (**C**) 42 days, (**D**) 70 days, and (**E**) 105 days later starting Kampo treatment.

Although, like Case 1, this case 2 was diagnosed as having a red tongue at her initial consultation, the changes in tongue color were more pronounced in Case 2. As shown in Figure 5 at the initial consultation, the patient's tongue was assessed as a red tongue between 14 days later and 70, the tongue showed a partial improvement but was still considered slightly red (Figures 5B,D). At 105 days of treatment, the tongue was judged to be a light red tongue, which is regarded as the normal tongue color (Figure 5E). The *ΔE*_00_ value of the tongue edge at 14, 42 and 70 days of treatment were 4.39, 5.22, and 4.99, respectively, indicating color changes that were perceptible to the human eye. Moreover, the *ΔE*_00_ value at 105 days later, when the tongue was assessed as light red, reached 10.08, indicating a readily noticeable and substantial color difference. These changes in *ΔE*_00_ values were consistent with the tongue color changes observed visually in the patient's tongue images.

## 4 Discussion

**　**Given the need for objective and reproducible methods of documenting tongue findings, the two cases illustrate the potential of TIAS for longitudinal documentation and quantitative comparison of tongue color. In particular, TIAS enabled subtle changes in tongue color to be quantified and compared over time, including changes that may be difficult to appreciate by visual observation alone. Such objective longitudinal records may assist physicians in clinical assessment by allowing comparison with previously recorded tongue findings without relying solely on memory or subjective impressions. However, these cases do not establish the diagnostic validity of TIAS or demonstrate that changes in tongue color directly reflect treatment efficacy.

Both of the cases presented herein were diagnosed as red tongue at the patient's initial consultations. When the initial consultation and second tongue images were compared, Case 2 showed a *ΔE*_00_ value of 4.39 at the tongue edge (Figure 5B), indicating a color difference that was perceptible to the human eye. In contrast, the *ΔE*_00_ at the tongue edge of case 1 was 1.65 (Figures 4A,B), representing a subtle color change that was difficult to perceive visually. In clinical practice, tongue color changes as subtle as those observed in case 1 are unlikely to be reliably assessed through medical record documentation or physicians’ visual memory.

Although smartphones and other digital photography devices can be used to record tongue images, they are often equipped with functions that automatically adjust brightness and color during or after image acquisition (35, 36). It is also difficult to reproduce uniform lighting conditions when using a digital camera or smartphone alone. These devices may therefore not be suitable for accurately recording longitudinal changes in tongue findings. However, TIAS is designed to block external light and acquire images that are always under uniform illumination conditions, enabling standardized recording and quantitative assessment of tongue characteristics. Because tongue images obtained with TIAS can provide consistent and objective information over time, we propose that TIAS may serve as a clinically useful tool to support physicians, pharmacists, and acupuncturists in the longitudinal assessment of tongue findings. In addition, by enabling tongue color assessments with reduced bias, TIAS may facilitate more objective documentation and comparison of changes observed during treatment. By reducing reliance on memory and subjective impressions when comparing tongue findings over time, TIAS may support more consistent clinical assessment and, ultimately, contribute to improved patient care.

An investigation of tongue characteristics over a 45-week period in patients with non-small cell lung cancer reported the usefulness of the continuous observation and quantitative assessment of tongue color (37). The investigation also indicated that early-stage changes in tongue color differed between responders and non-responders to treatment, suggesting that the monitoring of tongue color changes during the early phase of treatment may serve as a noninvasive prognostic biomarker. However, physiological and pharmacokinetic conditions differ between cancer patients and non-cancer patients. Although the usefulness of quantitative tongue color assessment has also been reported in various conditions, including upper gastrointestinal symptoms, diabetes mellitus, and cardiovascular diseases (4–8), these studies were investigations that compared tongue characteristics between patients and control groups at a single time point. We have found no published quantitative examination of temporal changes in tongue color in non-cancer patients. The two cases reported herein are non-cancer patients, and the use of TIAS enabled us to document longitudinal changes in tongue color alongside changes in the patients’ subjective symptoms. The two presented cases suggest that subtle longitudinal changes in tongue color may occur during the clinical course of treatment even in patients with common non-cancer-related symptoms. TIAS may facilitate the objective and quantitative documentation of such changes, which may provide information to assist physicians in clinical assessment during follow-up. However, these two cases do not establish that changes in tongue color directly reflect treatment efficacy; and further studies are required to determine the clinical significance of such changes.

The red tongue observed in case 1 and 2 is considered indicative of the presence of heat. It has been reported that high temperature and high humidity markedly enhance redness in the tongue color of patients with diabetes mellitus (38). Several mechanisms may contribute to increased tongue redness; for example, elevated body temperature may activate the hypothalamic–sympathetic nervous system, leading to capillary vasodilation in the tongue mucosa through mediators such as catecholamines (39). Heat stress has been reported to induce mild erythrocyte extravasation and the accumulation of oxyhemoglobin within the tongue through mechanisms involving the activation of endothelial nitric oxide synthase (eNOS), the generation of superoxide anions (O₂⁻), and the formation of peroxynitrite anions (ONOO⁻) (40). The present study's two cases in our study were also suspected to exhibit heat symptoms, and the observed reduction in a values (chromaticity; redness) occurred alongside improvement in the patients’ subjective symptoms. Because neither patient had diabetes mellitus, no conclusions can be drawn regarding the relationship between tongue color changes and the underlying physiological mechanisms. However, quantitative assessment of subtle tongue color changes at a level imperceptible to the human eye may provide a non-invasive means of detecting physiological changes potentially related to microcirculation.

The tongue imaging and quantitative assessment provided by TIAS may also contribute to the education of practitioners who are beginners at learning tongue diagnosis, including physicians, pharmacists, acupuncturists and medical students. Because proficiency in tongue diagnosis requires extensive clinical experience, standardized and objective records of tongue findings may be particularly valuable for novice practitioners who have not yet acquired sufficient experience to reliably recognize and compare tongue characteristics. Traditional medicine is estimated to be used by approximately 80% of the population in developing countries and nearly half of the population in developed countries, making it an important therapeutic option worldwide (17). Among the traditional medical practices, systems that incorporate tongue diagnosis, such as traditional Chinese medicine and acupuncture, have been reported by the World Health Organization (WHO) to be among the most widely practiced forms of traditional medicine globally (41). Given this background, the education and training of healthcare professionals involved in diagnoses have become matters of considerable interest not only in East Asian countries but throughout the world. Although many books are available for learning tongue diagnosis, tongue images acquired under diverse photographic conditions can be a source of confusion for learners (14, 15, 42–44). Acquiring practical competence in tongue diagnosis through book study alone is challenging. If the clinical experiences of expert practitioners (such as those involved in the current cases) can be accurately documented using TIAS and provided together with clinical judgement, this approach may support the education of novice learners of tongue diagnosis in countries around the world.

There are several study limitations to address. To evaluate tongue images objectively and reproducibly, it is important to control the imaging conditions. The TIAS used in the present study was developed to meet the requirements of ISO 20498-1 (CTIS—Part 1: General Requirements) and ISO 20498-2 (CTIS—Part 2: Lighting Environment), including standardized control of the imaging environment. However, even when the imaging conditions are standardized, TIAS cannot accommodate inadaptable patients who are unable to stick out their tongues (e.g., patients with congenital difficulties). In addition, imaging results may be affected when the distance between the tongue and TIAS is excessive or when the patient's face or tongue is positioned at an angle during image acquisition. Although all patients were instructed regarding how to stick out their tongue in advance and practiced before tongue images were obtained, it is not possible to obtain optimal images for all patients.

We had selected the cases of two patients for whom longitudinal tongue images were obtained, and we examined the changes in tongue color in the context of Kampo medicine. The cases illustrate the potential usefulness of TIAS for the objective and quantitative documentation of longitudinal changes in tongue findings. The results of our study indicated that by enabling standardized and reproducible imaging and quantitative assessment, TIAS may help physicians maintain accurate clinical records and compare tongue findings over time without relying solely on visual memory or subjective impressions.

## 5 Conclusion

TIAS enables longitudinal tongue imaging under standardized conditions and facilitates the quantitative comparison of subtle color changes that are difficult to discern visually. By providing objective and reproducible records of tongue findings over time, TIAS may be a clinically useful tool for supporting healthcare professionals in their clinical assessment and longitudinal comparison of tongue findings.

## Data Availability

The raw data supporting the conclusions of this article will be made available by the authors, without undue reservation.
